# Atrial Fibrillation and Stroke. A Review on the Use of Vitamin K Antagonists and Novel Oral Anticoagulants

**DOI:** 10.3390/medicina55100617

**Published:** 2019-09-20

**Authors:** Alfredo Caturano, Raffaele Galiero, Pia Clara Pafundi

**Affiliations:** Department of Advanced Medical and Surgical Sciences, University of Campania “Luigi Vanvitelli”, Piazza Luigi Miraglia 2, IT-80138 Naples, Italy; alfredo.caturano@virgilio.it (A.C.); raffaele_ga@outlook.it (R.G.)

**Keywords:** NOACs, non vitamin K oral anticoagulants, atrial fibrillation, stroke, oral anticoagulation

## Abstract

Atrial fibrillation (AF) is the most common arrhythmia, ranging from 0.1% in patients <55 years to >9% in octogenarian patients. One important issue is represented by the 5-fold increased ischemic stroke risk in AF patients. Hence, the role of anticoagulation is central. Until a few years ago, vitamin K antagonists (VKAs) and low molecular weight heparin represented the only option to prevent thromboembolisms, though with risks. Novel oral anticoagulants (NOACs) have radically changed the management of AF patients, improving both life expectancy and life quality. This review aims to summarize the most recent literature on the use of VKAs and NOACs in AF, in light of the new findings.

## 1. Introduction

Atrial fibrillation (AF) is the most common arrhythmia, ranging from 0.1% in patients aged <55 years to >9% in octogenarian patients. One of the most important issues is represented by the 5-fold increased risk of ischemic stroke in AF patients [1].

Atria are excited in a chaotic, disorganized manner, with a frequency of activation variable from 400 to 650 beats/min. The atrioventricular node (AVN) receives much more impulses from the atrium than it is able to conduct, thus exercising a filter function which transmits a not excessively high number of beats to the ventricles. In fact, numerous impulses penetrate only partially into the AVN and then they are trapped inside.

The patient is often symptomatic at onset. The most common symptom is palpitation, but, in the case of the concomitant presence of an organic heart disease, the loss of effective atrial systole, as well as tachycardia, favor a hemodynamic decompensation. Less frequently, AF runs asymptomatic.

The diagnostic suspicion may already arise at the evaluation of the radial pulse and/or the cardiac auscultation, and then confirmed by an electrocardiogram (ECG) characterized by the absence of regular and morphologically similar atrial activation waves, with a totally irregular interval of the QRS complexes of ventricular activation.

AF treatment has 4 main approaches:

1. Heart rate control with either beta blockers (Bisoprolol, Metoprolol), non-dihydropyridine calcium antagonists (Verapamil, Diltiazem), digoxin (less used due to the possible risk of toxicity, especially in patients with renal insufficiency) or, as a last resort, Amiodarone;
2. Either electrical or pharmacological cardioversion with class antiarrhythmics III (Amiodarone, Ibutilide) or I-C (Flecainide, propafenone, in the absence of cardiac structural damage);
3. AF deletion through catheter ablation, either by acting on its trigger points or by altering the arrhythmogenic substrate. In either case, the risk of relapse still persists, especially during the first 6–12 months after the procedure;
4. The control of thrombo-embolic complications by using anticoagulants (novel oral anticoagulants (NOACs), vitamin K antagonists (VKAs), heparin).

A more in-depth analysis of the latter point, in fact, shows that the reduction of blood flow in the atrial chambers, caused by the reduced ventricular depletion (consequent to the reduction of diastolic time and the loss of atrial contraction, as well as, sometimes, the reduction of myocardial contractility secondary to tachycardia) makes more likely the formation of thrombi in the left atrium (LA), including the left atrial appendage (LAA).

The occurrence of this condition significantly increases when arrhythmia lasts for over 48 h, with an embolic thrombus risk increased even more significantly at the reestablishment of the sinus rhythm. A risk stratification in these patients may be estimated by using the CHA2DS2-VASc score, for which a score is assigned to each risk factor, finally providing a sum which represents the overall risk of stroke per year for the patients (Table 1).

## 2. Atrial Fibrillation (FA) Cardioversion and Anticoagulation

Current ESC guidelines for patients with AF, for less than 48 h, with a CHA2DS2-VASc score of either 0 in men or 1 in women, recommend the administration of heparin, a factor Xa inhibitor or a direct thrombin inhibitor, versus no anticoagulant therapy, without the need for post-cardioversion oral anticoagulation. Conversely, an AF for 48 h or more, needs an appropriate anticoagulation for at least 3 weeks or a negative transesophageal echocardiogram (TEE), followed by 4 weeks anticoagulation after cardioversion. In the case of a rescue cardioversion due to hemodynamic instability, anticoagulation should be initiated as soon as possible and continued for at least 4 weeks after cardioversion, unless contraindicated [2].

A recent meta-analysis comparing warfarin and novel oral anticoagulants (NOACs) on 7588 AF patients undergoing electric cardioversion (CV) showed overlapping risks of ischemic stroke, major bleeding, mortality and hemorrhagic stroke [3]. In this subset of patients, several real-world studies have confirmed a favorable clinical outcome [4,5,6,7,8].

Though an appropriate therapy, the risk of systemic embolism in elective cardioversion is still present. In fact, a transesophageal echocardiogram may highlight the presence of a thrombus in LA or LAA in 5% of patients, despite adequate anticoagulation with both vitamin K antagonists (VKAs) or NOACs [9]. Data from real-world studies have highlighted a similar incidence of LA thrombus before performing CV, both among the use of different NOACs and in the case of VKA treatment [10,11]. Additionally, the importance of practicing TEE in patients at high risk of LA/LAA thrombus (e.g., CHA2DS2-VASc score >3) has been pointed out [12].

The average stroke rate <1% makes it reasonable to assume a lower prevalence of thromboembolism during cardioversion or, maybe, that not every stroke is clinically diagnosed. Moreover, it is not surprising that patients with a very high-risk score for thromboembolism could be refractory to standard anticoagulation [12].

The use of NOACs compared to VKAs treatment has shown, both in trials and in real-world settings, a reduction in the timing to CV, with a consequent higher satisfaction of patients and cost savings for clinical facilities [13,14,15].

In patients in whom sinus rhythm has been restored, the same drugs used for cardioversion may be used to prevent arrhythmia relapses. Among these drugs, amiodarone has been shown to be the most effective antiarrhythmic, though not without long term side effects [16].

## 3. Oral Anticoagulation with Vitamin K Antagonists

VKAs were the first anticoagulants used in AF patients. Their discovery was completely random and dates back to the 1920s, when in the U.S., sweet clover was used to feed livestock, which was stored in silos. The fermentation of the clover produced bis-hydroxycoumarin. The anticoagulant effect of this by-product determined the consistent death of herds of cattle on farms in Wisconsin, due to hemorrhagic syndromes. The fear that warfarin could be excessively toxic to humans initially led to only being used as rat poison. The drug with the trade name of Coumadin was approved only in 1954, though the skepticism of the medical community remained until 1955, when President Eisenhower, struck by coronary artery disease (CAD), requested to be treated with the most powerful “antithrombotic” drug of the time.

VKAs (warfarin and acenocoumarol) are indirect anticoagulants, which interfere with the hepatic production of dependent vitamin K coagulation factors. The lag time between drug intake and pharmacological action varies between 3 and 7 days, the time required for activated coagulation factors to be deleted and/or exhausted. On the other hand, prothrombin time (PT) can be lengthened in a short time due to the inhibition of short-life coagulation factors, such as factor VII.

The dosage of oral anticoagulants, due to the individual variability of their pharmacokinetics and pharmacodynamics, should be established based on the determination of the International Normalized Ratio (INR), given by the ratio between the PT of each patient and the PT of a healthy subject. In the case of AF, INR must be maintained between 2 and 3 [2].

Vitamin K represents the antidote of dicoumarols in the case of major bleeding, but it can also be found in several vegetables (e.g., tomatoes, spinach, cabbages, turnip greens), as well as in some dairy and animal products. Therefore, a reduction of the intake of these foods is strongly recommended to improve the time in therapeutic range (TTR).

The use of VKAs is limited by the narrow therapeutic interval, which needs frequent monitoring, dose adjustments and attention to drugs interaction (e.g., nonsteroidal anti-inflammatory drugs (NSAIDs) may lead to hemorrhage due to pharmacokinetic interactions and to their antiplatelet effect) [2].

VKAs efficacy and safety have been established over time and all over the world by several studies [17,18], and currently represent the first-choice treatment in AF patients with rheumatic mitral valve disease and/or a mechanical heart valve prosthesis [19]. Conversely, the use of NOACs in AF patients undergoing valves replacement and transcatheter aortic valve replacement (TAVR) is only supported by few and limited data [1,20].

In a meta-analysis, patients under VKAs therapy showed a relative risk reduction of ischemic stroke of 67%, with no significant difference between primary and secondary prevention, and 25% of all-cause mortality rate compared to controls (either aspirin or placebo). Also, the risk of intracranial hemorrhage was mild [21].

The fact that antiplatelet agents may play a preventive role during AF has been investigated by several studies. For example, Lip et al. [22], in a meta-analysis, demonstrated a 22% relative reduction in the risk of thromboembolism in AF with AP monotherapy compared to placebo. In addition, the authors also showed a 36% risk reduction with warfarin compared to aspirin. Several studies have compared warfarin to AP monotherapy and dual AP therapy (aspirin + clopidogrel), with a lower effectiveness of AP therapy and either a similar or increased risk of bleeding [23,24,25].

Thus, the most recent ESC guidelines have discouraged a routine use of AP monotherapy for stroke prevention in AF patients [26].

The Garfield AF registry shows how the administration of AP monotherapy in newly diagnosed AF has slowed down over the years, though a consistent number of patients are still under treatment (about 20% of the 51,270 patients analyzed are under AP monotherapy with no indication) [27].

Furthermore, AF patients cannot be treated with indirect anticoagulants if they are pregnant or breastfeeding, if they have bleeding diathesis or in the case of invasive surgical procedures. In addition, in fragile and/or cardiac and/or hepatic insufficient patients, closer INR controls are required.

## 4. Novel Oral Anticoagulants (NOACs): A Future Already Present

Until a few years ago, as shown in the previous sections, anticoagulant therapy with VKAs represented, along with the use of low molecular weight heparin (LMWH), the only therapeutic aid to reduce thromboembolic risk [28].

NOACs selectively inhibit only one factor of the coagulation cascade: thrombin, in the case of dabigatran, or activated factor X (Xa), in the case of rivaroxaban, apixaban and edoxaban.

Their pharmacodynamics are predictable, with little variability even at the individual level and there are no relevant interactions with both food and drugs. The half-life is well defined, but its increase with age and with the reduction of the renal filtrate should always be considered.

Their action is fast and their effect quickly ends after interruption and, in either case, can be predicted based on a few easily calculable variables (mainly the time from the last dose taken, type of molecule, age and the glomerular filtrate).

These characteristics make the monitoring of the coagulative structure superfluous (and confounding). In this way, the induction of the anticoagulant effect is eased without having to resort to the administration of heparin [28,29]. Moreover, both the safety and efficacy of NOACs have been positively tested in a randomized clinical trial [30] and confirmed by several clinical real-world casuistries [1,31,32,33,34,35].

For this reason, in recent years, NOACs have become a valid alternative to VKAs to prevent stroke in AF patients and have emerged as the first choice, especially in patients who are new to anticoagulants.

It is of fundamental importance to remember how some specific subpopulations of AF patients cannot be treated with NOACs. Among these are the wearers of cardiac mechanical prostheses, patients with severe mitral stenosis on a rheumatic basis and patients with aneurysms [22]. However, subjects with biological valve prostheses, subjected to mitral valvuloplasty three months after implantation, and those with hypertrophic cardiomyopathy have been granted by the 2018 EHRA PRACTICAL GUIDE update and 2016 ESC guidelines, the possibility of using NOACs [22,36].

Four large phase III trials assessed the non-inferiority of NOACs compared to VKAs. The overall assessment of the findings from the four trials allowed for establishing how NOACs are able to, with respect to conventional VKAs therapy, further reduce the combined risk of stroke and embolic events by 19% and the risk of all-cause mortality.

The prescription of the most appropriate NOAC must be based on the knowledge of the clinical characteristics of each patient and of the pharmacological characteristics of the different NOACs.

**Figure 1:**
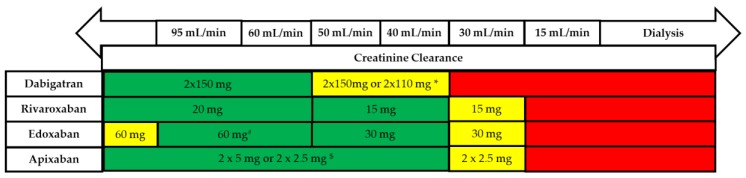
Use of non-vitamin K antagonists (VKAs) according to renal function. * 2 × 110 mg in patients at high risk of bleeding (per SmPc). ^#^ Other dose reduction criteria may apply (weight ≤60 kg, concomitant potent P-Gp inhibitor therapy). ^$^ 2 × 2.5 mg only if at least two out of three fulfilled: age ≥80 years, body weight ≤60 kg, creatinine ≥1.5 mg/dL (133 mmol/L). Orange arrows indicate cautionary use (dabigatran in moderate renal insufficiency, FXa inhibitors in severe renal insufficiency, edoxaban in ‘supranormal’ renal function). [36].

The recommended dosages for the treatment of AF patients are listed in Figure 1. To understand the profile of each NOAC, it is necessary to know the findings from the most important clinical trials which led to their registration.

The randomized open-label RE-LY clinical trial assessed the non-inferiority of dabigatran 150 mg bid (reduced to a 110 mg bid in elderly patients and in those with reduced renal function) compared to warfarin (INR 2 to 3) in AF patients. The study showed a statistically significant reduction in systemic stroke/embolism, hemorrhagic stroke and vascular mortality. The major bleeding rates were, instead, comparable. In addition, a significant reduction in the total number of bleedings, life-threatening bleeding for the patient and intracranial bleeding, as well as a statistically significant increase in gastrointestinal major bleeding with dabigatran 150 mg were observed [37].

In the ROCKET-AF double-blind randomized clinical trial, rivaroxaban was shown to be not inferior to Warfarin in the prevention of either stroke or systemic embolism, without significant difference between the two groups for overall mortality or differences between two drugs in the risk of major bleeding or major bleeding plus the clinically relevant ones. Even in the ROCKET-AF, however, a statistically significant increase in gastrointestinal major bleeding was observed [38].

Two trials, ARISTOTLE and AVERROES, instead assessed the efficacy and safety of apixaban 5 mg bid (reduced to 2.5 mg bid in elderly patients and in those with reduced renal function). Apixaban emerged statistically superior to Warfarin in the prevention of stroke and systemic embolisms, major bleeding, including intracranial ones, and no major clinically relevant ones, as well as in reducing all-cause mortality. Comparable outcomes emerged for major gastrointestinal bleeding [39,40].

Finally, edoxaban. The ENGAGE AF-TIMI 48 trial demonstrated the non-inferiority of edoxaban 60 mg vs. warfarin in preventing stroke or systemic embolic events, with a statistically significant reduction in hemorrhagic stroke, vascular mortality, major bleeding and the number of intracranial bleedings and a statistically significant increase in major gastrointestinal bleedings [41].

The meta-analysis of Dentali et al. states that all NOACs directly act on the final phase of the coagulation cascade, and therefore, differ from the VKAs mechanism of action [42].

In the prevention of stroke during AF, NOACs overall, compared to VKAs, significantly reduce (1) stroke and systemic embolism, (2) major bleeding, (3) intracranial bleeding, (4) cardiovascular and (5) global mortality.

Despite the several advantages of NOACs with respect to VKAs therapy, a careful decision-making process is required in each case to ensure the safety of the choice of one option over another.

As more findings emerge from clinical studies and real-world evidence, the use of NOACs is becoming increasingly varied, replacing VKAs therapy in many contexts as a safe, reliable and effective therapeutic approach [9,12,16,17,18,21,43]. However, VKAs still play an important role in countless contexts, including situations where NOACs are contraindicated [36].

At present, the difference between each NOAC depends on the preferences of the physician (evaluating the risk profile of each patient compared to that present in the groups treated in each study), the pros and cons of each molecule, and the costs. An indirect comparison between the four drugs can lead to the suggestion of which one would be preferred for each individual patient. A recent meta-analysis, including all of the four major clinical trials, showed that NOACs reduce ischemic events compared to warfarin in patients with AF, but at the cost of increased gastrointestinal bleeding [44]. The comprehensive results from all of these studies show a significant reduction in cases of stroke and systemic embolism (relative risk, RR, 0.81), mainly due to a reduction in hemorrhagic strokes (RR 0.49). There was also a small number of all-cause deaths, compared to warfarin, during follow-up (RR 0.90), though this did not affect myocardial infarction. Intracranial hemorrhages were less frequent with NOACs (RR 0.48), while gastrointestinal ones had a higher incidence (RR 1.25) [43].

In AF patients at high ischemic risk, who have undergone percutaneous coronary intervention (PCI) with stenting for acute coronary syndrome (ACS), dabigatran etexilate 110 mg twice daily versus VKAs, in association with DAPT (aspirin plus clopidogrel) showed a safer profile and a lower cumulative incidence of major bleeding, as well as a lower hospitalization rate for cardiovascular events in real-world settings [45,46].

## 5. Bridging Therapy

Perioperative management of AF patients receiving NOACs is an extremely sensitive issue. The strategy not to initiate the so-called “bridge therapy” is comparable to “bridge therapy” in terms of prevention of thromboembolic events, though it translates into a greater reduction in the risk of major bleeding. This requires a more in-depth consideration of the advantages of both pharmacokinetic and pharmacodynamic aspects of the different anticoagulation regimens in each individual patient [36].

Therefore, the management of patients who need to interrupt oral anticoagulant therapy (OAT) to undergo either surgery or invasive procedures is particularly complex and requires collaboration among the different medical figures. The American College of Cardiology Anticoagulation Work Group, in order to assess the current clinical practice, devised a specific survey. Several professionals, including cardiologists (in different sub-specialties), internists, gastroenterologists and orthopedists, were asked how to manage patients taking oral anticoagulant therapy (OAT), candidates for invasive procedures and surgical procedures [47].

With the advent of NOACs in most recent years, the decision-making process has become even more complicated, since guidelines on this issue only provide general recommendations. The BRIDGE study, published in the New England Journal of Medicine, attempted to address this issue. The study found that the no bridging strategy was inferior to the low molecular weight heparin bridging therapy for the prevention of thromboembolic events, while at the same time, it determined a reduction in the risk of major bleedings [48].

In particular, the BRIDGE study assessed how the different professional figures managed, in the common clinical practice, patients taking OAT as candidates for invasive procedures. From the findings of the study, the most frequently involved professional class was that of cardiologists. The study also showed that among the most commonly used parameters to identify patients with an increased risk of thromboembolic events during OAT interruption is the presence of a mechanical heart valve, a history of previous stroke or transient ischemic attack (TIA) and an elevated CHA2DS2-VASc score. With regard to this latter finding, it was emphasized that, frequently, this score is used in clinical practice to refer patients to the use of bridge therapy, though this approach has never been validated in this field. Despite many patients at low risk of thromboembolic events, that are referred to invasive procedures, being considered as low risk for bleeding without OAT interruption, the study showed that several doctors still prefer bridge therapy, exposing patients to a high risk of bleeding. Moreover, the variability in the choice of both dose and duration of parenteral anticoagulant therapy was also confirmed.

The study also underlined the problem of the management of patients on anticoagulant therapy with NOACs. A similar use of bridging therapy was observed for patients who were candidates for either surgical interventions or invasive procedures, treated with VKAs and with NOACs despite the extremely different pharmacokinetic characteristics of the drugs. In patients taking NOACs, however, in the case of an intermediate risk of thromboembolic events and in procedures with a low risk of bleeding, bridging therapy was used infrequently. Conversely, the use of parenteral anticoagulant therapy in high-risk patients treated with NOACs subjected to procedures with a higher risk of bleeding, requiring the interruption of anticoagulant therapy for a long period, has remained uncertain [49].

One of the drugs usable in the case of urgent procedures in subjects treated with dabigatran, who either had severe bleeding or required an urgent procedure, is the idarucizumab monoclonal antibody, studied in a trial of 503 patients, in the RE-VERSE AD study. Idarucizumab has received full FDA approval [50]. In addition, Andexanet alfa, a genetically modified and recombinant protein designed to serve as an antidote against direct factor Xa inhibitors, has also been reported to reverse the effects of rivaroxaban and apixaban and was approved according to the FDA’s accelerated approval process, based on the effects in healthy volunteers [51].

Furthermore, in a special subpopulation of patients undergoing coronary angiography with or without PCI, a meta-analysis by Kowalewski et al. showed a comparable safety of uninterrupted (UAC) and interrupted OAT (IAC). This safety also appeared higher in the case of IAC with bridging [52].

## 6. Anticoagulant Therapy: An Upcoming Challenge

AF is commonly diagnosed in the setting of active malignancy [53]. Cancer is associated with the hypercoagulable state, with an increased risk of thromboembolism, regardless of the CHA2DS2-VASc score [54]. Moreover, these patients, in particular the ones affected by either primary or metastatic intracranial tumors or hematological malignancies, also present an increased risk of bleeding. Other important issues should also be taken into account, such as drug–drug interaction with cancer treatment, changes in renal and hepatic function, dietary and nutritional status, chemotherapeutic toxicity and disease state. All these conditions may determine a fluctuation of INR values.

Up until now, VKAs have represented the gold standard in long term treatment. However, this class of drugs is burdened by the need to maintain the INR at target. In the last few years, with the advent of NOACs, several studies have assessed the safety and efficacy in this specific population [32,55,56,57,58]. Nevertheless, the limited sample size and the wide spectrum of malignancies render it necessary to conduct further in-depth studies.

Thus, anticoagulation with both NOACs and VKAs for AF related thromboembolism in patients affected by malignancies is challenging.

## 7. Conclusions

In conclusion, given the extreme complexity of this scenario, which involves multiple professional figures, it would be worthwhile establishing standardized protocols and research models oriented towards the development of clinical pathways. In this way we could improve the management of patients under OAT, candidates for interventions surgical and invasive procedures, especially in light of the new commercial oral anticoagulants.

## Figures and Tables

**Table 1:** Risk stratification of stroke by the CHA2DS2-VASc score [2].

Risk Factors	Score	CHA2DS2-VASc Score	Stroke Risk Per Year
Congestive Heart Failure	1	0	0%
LV Dysfunction	1	1	1.3%
Hypertension	1	2	2.2%
Age ≥ 75 years	2	3	3.2%
Diabetes Mellitus	1	4	4.0%
Stroke/TIA/Thromboembolism	2	5	6.7%
Vascular Disease	1	6	9.8%
Age 65–74	1	7	9.6%
Female	1	8	6.7%
Total	9	9	15.2%

LV: Left Ventricle, TIA: Transient Ischemic Attack.
